# Treatment of congenital pulmonary airway malformation with rare high cystic volume ratio: A case report and literature review

**DOI:** 10.1097/MD.0000000000036249

**Published:** 2023-11-24

**Authors:** Miao Huang,, Yun-Hui Gong

**Affiliations:** a Department of Gynecology and Obstetrics, West China Second University Hospital, Sichuan University, Chengdu, Sichuan Province, China; b Key Laboratory of Birth Defects and Related Diseases of Women and Children, Ministry of Education, West China Second Hospital, Sichuan University, Chengdu, Sichuan Province, China.

**Keywords:** case report, congenital pulmonary airway malformation, pregnancy complications, prenatal care, respiratory tract diseases

## Abstract

**Rationale:**

Congenital pulmonary airway malformation (CPAM) is a rare congenital dysplastic malformation and accounts for 25% of congenital lung lesions. Commonly, it is diagnosed prenatally in ultrasound. The CPAM volume ratio (CVR) is a well-recognized predictor of fetal prognosis, and when the CVR is >1.6 cm^2^, the fetus is very likely to develop hydrops and even intrauterine deaths. However, the association of CVR with a wide range of complications and neonatal prognosis is unclear.

**Patient concerns:**

Cystic lesions in the right thorax of the fetus detected by ultrasound at 19 weeks of gestation, with a CVR of 0.88 cm^2^. The CVR grew progressively with increasing gestational weeks, reaching a maximum of 5.2 cm^2^ at 35 gestational weeks. However, there were no complications with the fetus other than polyhydramnios.

**Diagnosis:**

Imaging and pathological findings confirmed the diagnosis of CPAM.

**Interventions:**

During pregnancy, a multidisciplinary team was involved in the management and the prenatal visits increased to weekly from 31 weeks of gestation. During the cesarean section, neonatologists and pediatric surgeons were present for timely evaluation of newborns. The neonate was admitted to the neonatal intensive care unit for monitoring immediately after birth and underwent thoracoscopic right lower lobectomy at 57^th^ days old.

**Outcomes:**

The neonate recovered without any respiratory symptoms and no abnormality on chest computed tomography (CT) at the 3-month postoperative follow-up.

**Lessons:**

During pregnancy, in addition to monitoring CVR, a multidisciplinary team should join in the management of CPAM patients. And as for the fetus with increased CVR, a closely monitoring after birth is necessary even if the general condition of the pregnancy is well. In particular, timely intervention should be made at the onset of respiratory symptoms.

## 1. Introduction

Congenital pulmonary airway malformation (CPAM) is a rare developmental congenital anomaly of the lungs with an incidence of 0.94 per 10,000 live births and is characterized by lung cysts.^[1]^ The cysts are formed due to the focal arrest of lung development and usually can be detected by prenatal ultrasound. The compression caused by the lesions can result in complications such as fetal heart dysfunction, polyhydramnios, hydrops, and even intrauterine death. Thus, the CPAM volume ratio (CVR) is often used to predict fetal prognosis. It is calculated by the volume of the CPAM (width × height × length × 0.523) divided by the head circumference. CVR >1.6 cm^2^ is generally considered to indicate a high risk of fetal hydrops.^[2]^ However, the association of CVR with a wide range of complications and neonatal prognosis is unclear. Based on a review of the literature, we believe this case presents the largest CVR (5.2 cm^2^) of CPAM without fetal hydrops. However, the neonatal presented respiratory symptoms a few days after birth, which supported that the continued increase of CVR in the third trimester may lead to an adverse neonatal outcome.

## 2. Case presentation

**Figure 1.**
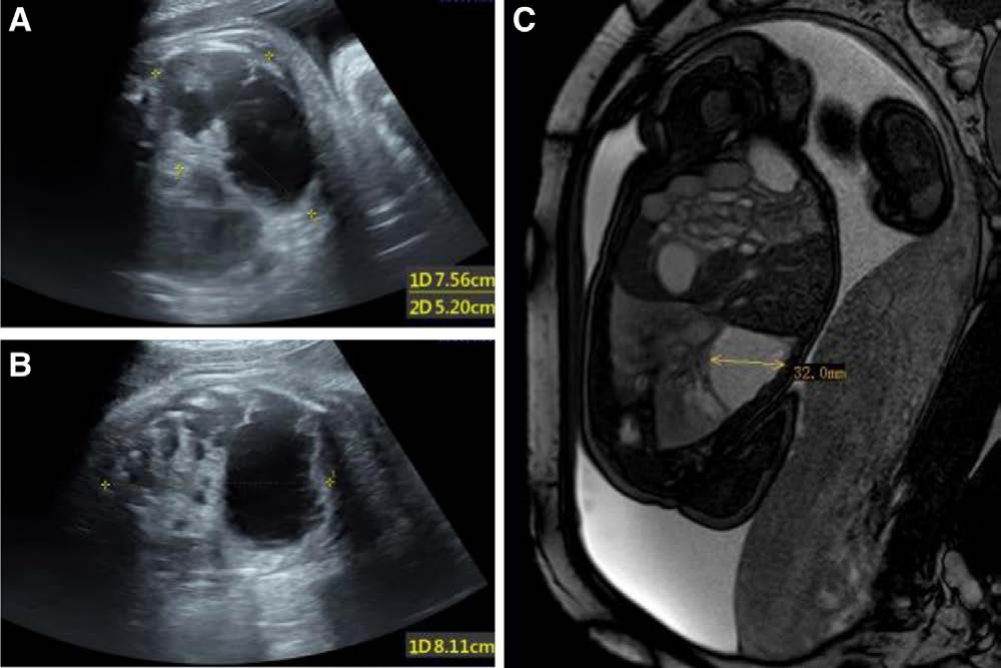
(A) and (B) Antenatal ultrasound at 35 wk gestational. (C) MRI of the uterus and the fetus at 34 wk gestational. MRI = magnetic resonance imaging.

A 43-year-old woman, G3 P1011, with diet-controlled gestational diabetes mellitus visited our hospital for routine antenatal care. An ultrasound scan at 19 + gestational weeks revealed several cystic lesions with different sizes in the right lung of the fetus, and the largest cyst was about 1.6 cm in diameter. Thus, CPAM with a CVR of 0.88 cm^2^ was considered. Because the chromosome microarray analysis, fetal structures, and fetal echocardiograph were normal, the pediatric surgeon and obstetrician suggested a regular review. At 31 gestational weeks, ultrasound detected polyhydramnios with a deepest vertical pocket of amniotic fluid of 8.8 cm and amniotic fluid index of 21.2 cm. Because the cystic lesions increased in size with pregnancy (Table 1), a multidisciplinary team including an obstetrician, a neonatologist, a pediatric surgeon, a geneticist, and a radiologist was involved. The prenatal visits increased to weekly but prenatal intervention was not recommended because of the stable condition of the fetus. Magnetic resonance imaging (MRI) was performed at 34 gestational weeks (Fig. 1). It revealed that cysts varying in size replace most of the normal right lung tissue. In addition, no definite blood supply from the systemic circulation to the largest cyst (3.9 cm × 3.4 cm) was observed. The last ultrasound scan at 35 gestational weeks, as shown in the Figure 1, revealed a CVR of 5.2 cm^2^ and mediastinal displacement. The multidisciplinary team suggested terminating the pregnancy prematurely because of lesions progression and complete placenta previa. At 36 + 2 gestational weeks, a repeat cesarean section was conducted following 4 doses of dexamethasone (6 mg i.m.). The neonate, a girl, weighing 3.16 kg with Apgar scores of 9 and 10 at 1 and 5 minutes respectively was born. She was immediately inspected by the pediatric surgeon and neonatologist and be transferred to neonatal intensive care unit.

**Table 1:** Ultrasound index changing during pregnancy.

Gestational wk	19	25 + 5	31 + 3	33 + 4	35 + 4
CVR (cm^2^)	0.88	1.62	2.16	4.6	5.2
DVP of amniotic fluid (cm)	4	6.1	8.8	9.0	12.6
AFI (cm)	/	/	21.2	22.9	34

AFI = amniotic fluid index, CVR = cystic volume ratio, DVP = deepest vertical pocket.

**Figure 2.**
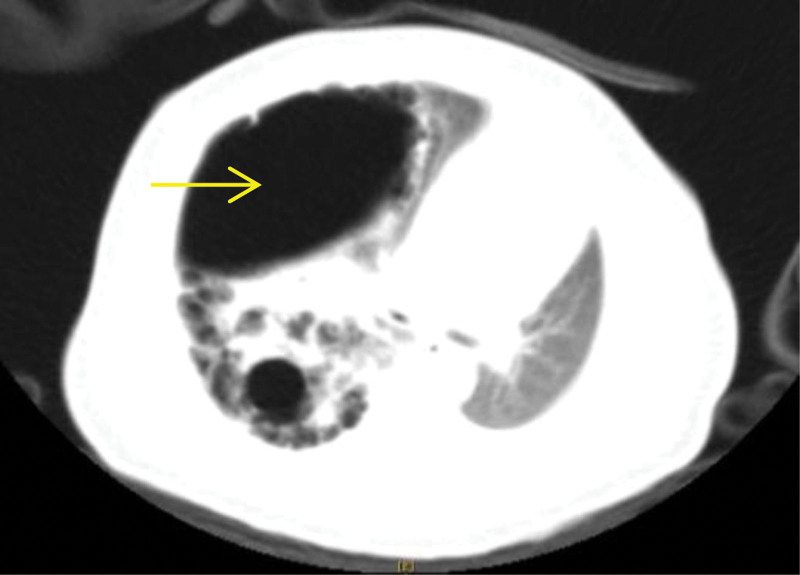
CT at 2 days after birth suggested cystic lesions of variable size in the right lung, mediastinum shift, and compression of the left lung and heart. CT = computed tomography.

Enhanced- and high-resolution computed tomography (CT) scan (Fig. 2) of the chest after birth revealed multiple cystic lesions in the lower lobe of the right lung, the largest cyst was about in size 4.7 cm × 4.1 cm with an air-fluid level. And the mediastinum showed a left shift. The neonate presented with respiratory distress and pneumonia 4^th^ days old, thus, anti-infective therapy and thoracic drainage was conducted. At 57^th^ days old, the infant symptoms were improved and a thoracoscopic right lower lobectomy was conducted. The pathological diagnosis was CPAM with infection. A CT scan 3 months after the surgery showed that the remaining right lung was well-developed with no pleural effusion. And there was no dyspnea, pneumonia, or other signs and symptoms of lung disease.

## 3. Discussion and conclusions

According to histologic features, CPAM can be divided into 4 types. Type 0 is essentially a tracheobronchial defect. Type 1 and type 4 are cysts up to 10cm, but their pathological manifestations are different. Type 2 is multiple small cysts (<2cm) and solid pale tumors. Type 3 is almost solid tissue with small air spaces separating the bronchiolar structures.^[3,4]^ However, compared with the histologic type, the size of the lesions is more correlated with the prognosis.

With the ongoing improvement of technology, most patients with CPAM are diagnosed during the fetal period by ultrasound scan. Although it is often confused CPAM with other congenital lung lesions, such as bronchopulmonary sequestration that can coexist with CPAM,^[5]^ most congenital lung lesions display a similar natural history.^[6]^ Therefore, there is no great difference in pregnancy management. If CPAM is suspected, chromosomal and other structural abnormalities should be excluded to confirm the continuation of pregnancy. The subsequent management of pregnancy should be done by regular ultrasound examination to monitor changes in the lesions and other complications. Compression caused by the lesions may lead to fetal hydrops and adverse effects on the development of the lungs and heart. Last decades, the indicators obtained by ultrasound are used to predict fetal prognosis in CPAM patients, including the CVR, lung-to-thorax transverse ratio, and mass-thorax ratio.^[7]^ Other researchers suggested that measures reflecting the overall physiologic impact of the lesion on the adjacent organs can assist in predicting prognosis, such as cardiomediastinal shift angle.^[7,8]^ Among those indicators, CVR is the most frequently used. Formerly, patients are usually believed to suffer from fetal hydrops when CVR > 1.6 cm^2^. But the correlation between CVR and neonatal respiratory morbidity is not well enough. According to the cases in Table 2, except for 1 case, all other live birth cases with reported high CVR had symptoms of hydrops and need prenatal interventions. That only fetus who did not develop hydrops had the lowest initial CVR of all the cases, and the lesion grew slower than in another case with the same initial value.^[9]^ The initial CVR of our patient (0.88 cm^2^) was lower than it of all cases in Table 2, and there was no hydrops even if the maximum CVR (5.2 cm^2^) was much >1.6 cm^2^. A retrospective study suggested that a CVR<0.56 cm^2^ on the initial ultrasound is predictive of a good outcome, but a CVR>0.56 cm^2^ is less useful for the prediction of adverse outcomes.^[10]^ Another recent systematic review reported that a threshold as low as 0.4 cm^2^ is more useful for predicting a broader range of fetal outcomes.^[2]^ To summarize the above, a lower initial CVR, rather than a higher maximum CVR, may have greater predictive value for fetal prognosis.

**Table 2:** case reports of live birth CPAM.

Paper	Initial value of CVR (cm^2^)	CVR-max (cm^2^)	Ascites	Polyhydramnios	Edema	Other complications	Prenatal intervention†
Brenda Hui Yan Law et al^[1]^	(2.1 × 1.0 × 1.3)	(7.9 × 6.2 × 4.2)	Y*	N*	N	Pleural effusion	a
Frances L. Lee et al^[2]^	3.1	7.8	Y	Y	N	Pericardial effusion	b
	3.4	4.8	Y	Y	Y	Pleural effusion	b and c
	3.9	3.9	Y	N	Y	N	b
Serah E. sheppard et al^[3]^	3.8	3.8	Y	Y	Y	Pleural effusion, Nevus comedonicus syndrome	c
Anastasiya holubyeve et al^[4]^	(3.01 × 2.06)	3.17	Y	Y	Y	Mosaic Klinefelter syndrome	a and c
Jinxi Huang et al^[5]^	1.8	2.99	N	N	N	N	N
M. Isnard et al^[6]^	1.8	4.6	Y	Y	N	N	a
Hayase Nitta et al^[7]^	1.95	2.6	Y	Y	Y	N	a

CVR = cystic volume ratio.

* Y: yes, N: no.

† a: Thoracoamniotic shunt, b: percutaneous sclerotherapy, c: steroid administration.

1. Law, B.H., et al, *Refractory tension pneumothorax as a result of an internally displaced thoracoamniotic shunt in an infant with a congenital pulmonary airway malformation*. BMJ Case Rep, 2016;2016.

2. Lee, F.L., et al, *Treatment of congenital pulmonary airway malformation induced hydrops fetalis via percutaneous sclerotherapy*. Fetal Diagn Ther, 2012;31(4):264–8.

3. Sheppard, S.E., et al, *Further delineation of the phenotypic spectrum of nevus comedonicus syndrome to include congenital pulmonary airway malformation of the lung and aneurysm*. Am J Med Genet A, 2020;182(4):746–754.

4. Holubyeva, A., et al, *Congenital pulmonary airway malformation associated with mosaic Klinefelter syndrome*. J Clin Ultrasound, 2020;48(2):121–124.

5. Huang, J., et al, *Thoracoscopic lobectomy for a 4-day-old neonate with a large congenital pulmonary airway malformation: a case report*. J Cardiothorac Surg, 2020;15(1):159.

6. Isnard, M., et al, *Successful intrauterine therapy for congenital cystic adenomatoid malformation of the lung. A case report*. Fetal Diagn Ther, 2007;22(5):325–9.

7. Nitta, H., et al, *Fetal Thoracoamniotic Shunting in a Case of Congenital Pulmonary Airway Malformations with Hydrops Fetalis*. AJP Rep, 2017;7(3):e185-e187.

However, there is a lack of evidence on the relationship between changes in CVR during pregnancy and prognosis. Past studies found that the fastest rate of growth in CPAM usually occurs between 20 and 25 gestational weeks, and most of them regress or even resolve after 28 gestational weeks.^[5,11]^ But in our case, the lesions continue to grow until deliver. And there was no significant correlation between lesion growth rate and hydrops severity in cases from Table 2. Even so, we increased the frequency of visits to once a week from 31 gestational weeks because of a rapid increase in the volume of lesions and the appearance of polyhydramnios.

In the case of fetal hydrops, prenatal intervention is necessary, otherwise intrauterine fetal death is likely to occur. Current prenatal interventions include maternal steroid administration, percutaneous sclerotherapy, thoracoamniotic shunting, and fetal surgery. The steroid administration may restrain the lesions’ growth rate, but the exact usage is debatable, and the effect is variable. Percutaneous sclerotherapy is a minimally invasive therapy compared with fetal surgery, but it may cause fetal demise.^[12]^ Thoracoamniotic shunting is another minimally invasive therapy and the most appropriate treatment for patients presenting with macrocystis. It also had side effects, including rupture of membranes, preterm labor, chorioamnionitis, and repeat shunting secondary to shunt occlusion or displacement. Fetal surgery for CPAM has a limited improvement in pregnant prognosis and is associated with maternal risk. The cases in Table 2 do not show which prenatal intervention is better. Therefore, clinicians need to consider their technical proficiency, the patient condition, and other specific factors to design an individual treatment plan for each patient. In our case, although the patient had polyhydramnios, no other complications were present. And repeating ultrasound scans showed a satisfactory fetal growth rate and normal cardiac function. Therefore, a multidisciplinary consultation was necessary. Compared with the risks of prenatal intervention, close observation, and increasing hospital visits were recommended.

CPAM cannot be used as an indicator to decide the timing or the method to terminate the pregnancy. At birth, the neonatology team should be present to evaluate whether resuscitation or emergency surgery is required for the newborn. Furthermore, the treatment can differ after birth depending on the presence of symptoms in the neonates. Surgery is the standard treatment for those with symptoms, whereas its requirement in asymptomatic neonates is still controversial.^[13]^ Some researchers suggest that all patients with CPAM require surgery because they believe that bronchioloalveolar carcinoma and rhabdomyosarcoma are associated with CPAM malignancy.^[14]^ Furthermore, patients may suffer from repeated lung infections, bronchiectasis, and lung abscesses during growth. And elective surgery has a better prognosis and fewer complications, compared with emergency surgery.^[5]^ A retrospective study revealed a good prognosis related to surgery; the pathological examination helped confirm the diagnosis.^[15]^ However, certain studies contradict active surgical treatment because there is no reliable supporting data related to the incidence of malignant changes and respiratory symptoms seldom appear in original asymptomatic CPAM patients.^[16]^ Therefore, patients should be followed up by regular chest CT scans, and early surgery should not be performed.^[17]^ In our case, the CT scan revealed multiple cystic lesions in the lower lobe of the right lung with an air-fluid level. And symptoms of pneumonia appeared 4 days after birth. Therefore, the pediatric surgeon placed a chest drain in the largest cyst to remove fluid and relieve inflammation. Elective surgery was performed after the neonate general condition became stable. No abnormality in the heart and lung functions was reported at the follow-up. Moreover, no residual lesions were detected 3 months after the surgery.

For CPAM detected prenatally, management and intervention during pregnancy are more complex than after birth. Preciously, CVR > 1.6 cm^2^ was used to predict the development of fetal hydrops. Based on recent literature and experience in this case, a lower initial CVR may be more valuable in predicting a positive fetal prognosis. In addition to monitoring CVR, a multidisciplinary team should join in the management of CPAM patients. And as for the fetus with increased CVR, a closely monitoring after birth is necessary, and timely intervention should be made at the onset of respiratory symptoms.

## Author contributions

**Funding acquisition:** Yun-Hui Gong.

**Investigation:** Miao Huang.

**Project administration:** Yun-Hui Gong.

**Writing – original draft:** Miao Huang.

**Writing – review & editing:** Yun-Hui Gong.
